# Predictors of Mortality, Drug Resistance, and Determinants among Carbapenem-Resistant *Enterobacterales* Infections in Chinese Elderly Patients

**DOI:** 10.1155/2024/5459549

**Published:** 2024-08-28

**Authors:** Yufei Zhang, Chengyun Zou, Jie Qin, Muyi Li, Xing Wang, Tian Wei, Haiying Wang

**Affiliations:** ^1^Department of Clinical Laboratory, Yueyang Hospital of Integrated Traditional Chinese and Western Medicine, Shanghai University of Traditional Chinese Medicine, Shanghai 200437, China; ^2^Department of Clinical Laboratory, Shanghai Fifth People's Hospital, Fudan University, Shanghai 200240, China; ^3^Department of Laboratory Medicine, Shanghai Children's Medical Center, Shanghai Jiaotong University School of Medicine, Shanghai 200127, China

## Abstract

Elderly patients with carbapenem-resistant *Enterobacterales* (CRE) infections represent considerable mortality rates. But data on the risk factors for the death of elderly patients following such infection remain limited. We aimed to assess the clinical outcomes, identify mortality-associated risk factors, and determine the antibiotic resistance and resistance genes of isolates for these patients. Hospitalized patients aged ≥65 years with CRE infection from January 2020 to December 2020 were retrospectively reviewed. Isolates identification and molecular characterization of CRE were carried out. Logistic regression analysis was applied to assess the potential factors associated with mortality. Of the 123 elderly patients with CRE infection included in our study, the all-cause mortality rate was 39.8% (49/123). The most prevalent pathogen was carbapenem-resistant *Klebsiella pneumoniae* (CRKP, 116 of 123). The overall rates of multidrug-resistant (MDR) and extensively drug-resistant (XDR) were 100.0% and 66.7%. All CRE isolates exclusively harbored a singular variant of carbapenemase gene, such as *bla*_KPC−2_, *bla*_IMP−4_, *bla*_NDM−5_, or *bla*_OXA−48_, while 98.4% of isolates harbored more than one *β*-lactamase gene, of which 106 (86.2%) isolates harbored *bla*_CTX−M_, 121 (98.4%) isolates harbored *bla*_TEM_, and 116 (94.3%) isolates harbored *bla*_SHV_. Multivariable logistic regression analysis revealed that mechanical ventilation (adjusted odds ratio (AOR) = 33.607, 95% confidence interval (CI): 4.176-270.463, *P* < 0.001), use of tigecycline during hospitalization (AOR = 5.868, 95% CI: 1.318-26.130, *P* = 0.020), and APACHE II score (AOR = 1.305, 95% CI: 1.161–1.468, *P* < 0.001) were independent factors associated with increasing the mortality of patients with CRE infection, while admission to intensive care unit (ICU) during hospitalization (AOR = 0.046, 95% CI: 0.004–0.496, *P* = 0.011) was a protective factor. CRE-infected elderly patients with mechanical ventilation, use of tigecycline during hospitalization, and high APACHE II score were related to poor outcomes. The isolates carried various antibiotic genes and presented high antibiotic resistance. These findings provide crucial guidance for clinicians to devise appropriate strategies for treatment.

## 1. Introduction

Over the last two decades, a significant increase in the prevalence of carbapenem-resistant *Enterobacterales* (CRE) has been observed worldwide [1–3]. In China, the rates of imipenem-resistant or meropenem-resistant *Escherichia coli* (*E. coli*) and *Klebsiella pneumoniae* (*K. pneumonia*) have escalated from 1.1% or 1.4% and 3.0% or 2.9% in 2005 to 1.8% or 2.0% and 23.1% or 24.4%, respectively, in 2021 [4]. The limited availability of antimicrobials has posed an obstacle to clinicians and clinical microbiologists in managing CRE, which has been identified as an antibiotic resistance threat by US and international health agencies [5].

The mechanisms underlying carbapenem resistance in *Enterobacterales* bacteria are rather intricate. It was attributed to the production of carbapenemases, deletion or mutation of outer membrane protein, overexpression of efflux pumps, or the alteration of penicillin-binding protein and biofilm components, which can result in the degradation of antibiotics, prevention of antibiotic penetrating into bacterial cells, alterations in antibiotic binding targets, or increased excretion of carbapenems [6]. Nevertheless, it is widely recognized that the primarily facilitated resistance lies in the dissemination of genes encoding carbapenem-hydrolyzing enzymes. These enzymes include serine carbapenemases, which consist of class A enzymes such as KPC, SME, NMC, IMI, and GES, as well as class *D* enzymes such as OXA. In addition, resistance can also result from the presence of metallo-*β*-lactamases such as VIM, NDM, IMP, SPM, GIM, and SIP [7–9]. On top of that, the production of extended spectrum *β*-lactamase (ESBL) is also a crucial factor contributing to carbapenem resistance in CRE. According to the previous study, the occurrence of CRE could be exacerbated by the production of ESBLs [10]. And carbapenem antibiotics was not be hydrolyzed by ESBL, so that it was served as the primary therapeutic approach for treating infections induced by ESBL-producing bacteria [11]. The prevalent utilization has prompted the emergence of CRE. To date, there have been over 500 *β*-lactamase documented in *Enterobacterales* strains. Of these, the CTX-M, TEM, and SHV *β*-lactamases are the predominant variants and are known to be promiscuous and transmissible across diverse epidemiological environments [7, 12, 13].

A growing pile of evidence suggests CRE infection is associated with considerable mortality, ranging from 10% to 90% [14], especially in bloodstream infection, intensive care unit, and solid organ transplantation [15]. In addition, older age was considered as associated with death in CRE-infected patients [16]. However, while the risk factors for CRE infection have been extensively studied, considerably less is known about the determinants of adverse outcomes resulting from comprehensive assessment in elderly patients with CRE infection [17–19]. Therefore, the aim of this study was to assess the clinical outcomes, identify risk factors associated with mortality, determine the antibiotic resistance and relevant genes of isolates for elderly patients with CRE infection in China.

## 2. Materials and Methods

### 2.1. Study Setting and Population

We designed a retrospective, non-interventional study at Yueyang Hospital of Integrated Traditional Chinese and Western Medicine, a 1200-bed tertiary teaching hospital for Shanghai University Traditional Chinese medicine in Shanghai, China, from January to December 2020. The Research Ethics Committee of Yueyang Hospital of Integrated Traditional Chinese and Western Medicine, Shanghai University of Traditional Chinese Medicine, evaluated and approved this study (approval number: (2021)060). By reviewing the hospital information system, hospital inpatients, aged ≥65 years, diagnosed with CRE nosocomial infection according to the criteria issued by the Ministry of Health in 2001 [20], with positive microbiological samples were recruited and not duplicated. To mitigate the effect of bias, patients with insufficient clinical data, surveillance or screening cultures, or subsequent episodes of the same patient were excluded. In addition, patients showing positive microbiological results but relevant clinical symptoms and abnormal laboratory tests were not presented would be identified as contamination or colonization of isolates and are also excluded. Based on the discharge diagnosis recorded on the cases, they were categorized into two groups to analyze the factors associated with mortality, patients who passed away during hospitalization were designated as the death group and the survivor group was comprised of patients who were discharged with favorable outcomes.

### 2.2. Data Collection

Clinical and epidemiological data were retrieved from medical records of all cases, including date of admission, demographics (age and gender), underlying medical conditions (cardiovascular and cerebrovascular diseases, chronic lung diseases, chronic kidney diseases, biliary tract diseases, solid tumors, diabetes, and hypertension), intervention therapy (surgery, mechanical ventilation, intravenous catheterization, gastric intubation, urinary catheterization, and sputum aspiration), antimicrobial therapy during hospitalization (glycopeptides, oxazolidinones, chloramphenicols, *β*-lactams/*β*-lactamase inhibitors, tigecycline, third- or fourth-generation cephalosporins, first- or second-generation cephalosporins, carbapenems, quinolones, and aminoglycosides), admission to intensive care unit (ICU), health care and previous antibiotic exposure within 30 days before admission, length of hospital stay (LOS), and outcomes (survivor or death) in the hospital period. Previous antibiotic exposure was defined as the reported intake of antibiotics for at least 48 hours within 30 days before hospitalization. The acute physiology and chronic health evaluation (APACHE II, at the time of discharge or death) scoring system and the Charlson Comorbidity Index score (CCIS, at admission) were recorded. Microbiological data, including specimen source, bacterial species, and the results of antimicrobial susceptibility testing (AST), were obtained by reviewing the hospital laboratory information system (LIS).

### 2.3. Isolates Identification and Antimicrobial Susceptibility Testing

All strains were isolated from clinical specimens and cultured on Columbia blood agar plate. And we employed a VITEK-2 compact system (bioMérieux, Marcy-l'Étoile, France) to perform the isolate identity. All isolates were further confirmed via the MALDI-TOF MS system (Bruker Daltonics, Billerica, USA). The VITEK-2 compact system was also used for antimicrobial susceptibility testing to further confirm whether *Enterobacterales* are CRE. CRE in this present study was interpreted on the basis of the 2020 Clinical and Laboratory Standards Institute (CLSI) breakpoints that an *Enterobacteriale* isolate was resistant to imipenem or meropenem (i.e., minimum inhibitory concentration (MIC) ≥ 4 *μ*g/mL) [21]. The relevant isolates were collected and stored at −80°C for further bacteriological and molecular characterization analysis.

Broth microdilution assay (BIO-KONT, Wenzhou, China) was performed against 13 different antibiotics, including imipenem, ceftazidime, aztreonam, ceftazidime-avibactam, tigecycline, cefepime, polymyxin B, piperacillin-tazobactam, amikacin, imipenem-avibactam, cefoperazone-sulbactam, meropenem, and levofloxacin, to determine the MICs of antimicrobial agents. *E. coli* ATCC 25922 was used as a quality control. The results were interpreted following the standards of the 2020 CLSI guidelines [21]. The breakpoint guidelines of the Food and Drug Administration (FDA) standard and the European Committee on AST (EUCAST) were applied to the interpretive criteria of polymyxin B and tigecycline, respectively [22, 23]. The breakpoints of cefoperazone and imipenem in CLSI were designated as ones of cefoperazone-sulbactam and imipenem-avibactam, respectively [24, 25]. Based on the result of broth microdilution assay, isolates were further identified as multidrug-resistant (MDR) and extensively drug-resistant (XDR) referring to the following criteria in *Enterobacterales*: MDR was defined as the state of being nonsusceptible to at least one drug in three or more antimicrobial classes, while XDR as nonsusceptible to at least one drug in all but two or fewer antimicrobial categories (i.e., bacterial isolates are sensitive to only one or two classes) [26].

### 2.4. Genotypic Detection of Carbapenemase and ESBL Genes

The boiling method was used to extract the genomic DNA as described previously [27]. All isolates were characterized by polymerase chain reaction (PCR) to investigate the genetic identification of carbapenemases comprising *bla*_KPC_, *bla*_OXA−48_, *bla*_IMP_, *bla*_NDM_, and *bla*_VIM_, as well as other *β*-lactamase genes including *bla*_SHV_, *bla*_CTX−M_, and *bla*_TEM_, according to previous studies [28–32]. The comprehensive description of the PCR process, as well as the primer sequences, can be found in the Supplementary Table 1. Subsequently, the PCR products were visualized by agarose (1%) gel electrophoresis. And the amplified positive PCR products were further confirmed by direct DNA sequencing (Sangon Biotech, Shanghai, China). The variants of carbapenemase and ESBL genes were determined by comparing nucleotide sequences with the existing sequences in the NCBI GenBank database by the BLAST program (https://blast.ncbi.nlm.nih.gov/Blast.cgi).

### 2.5. Multilocus Sequence Typing (MLST)

MLST was performed on all CRKP isolates as described in online databases to shed light on the genetic relatedness, and seven housekeeping genes (*phoE*, *pgi*, *tonB*, *rpoB*, *gapA*, *infB*, and *mdh*) were amplified and then sequenced by Sangon Biotech (Shanghai, China). This website (https://bigsdb.pasteur.fr/klebsiella/primers-used/) was used to compare the results to assign the sequence types (STs).

### 2.6. Statistical Analysis

The CCIS and APACHE II scoring systems were calculated. The statistical package SPSS version 26 (IBM SPSS Inc., Chicago, IL, USA) was conducted to analyze data, with *P* < 0.05 being the statistically significant threshold for all tests. The demographic, clinical, and molecular data were presented as median with interquartile range (IQR) for quantitative variables or the frequencies with percentages for qualitative variables. The comparison between groups was performed by the Mann–Whitney *U* test for continuous variables and with the Pearson chi-square test or Fisher's exact test for categorical variables. The statistically significant factors from the univariable analysis were further evaluated using the logistic regression analysis to identify the factors related to mortality. The crude odds ratios (CORs) with 95% confidence intervals (CIs) of the univariable analysis and adjusted odds ratios (AORs) with 95% CIs of the multivariable analysis were calculated.

## 3. Results

### 3.1. Sociodemographic Characteristics

**Figure 1:**
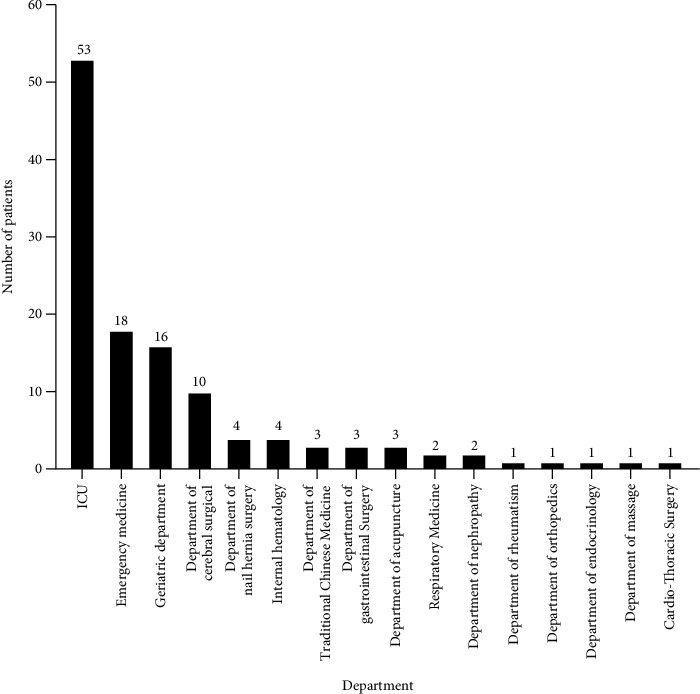
The department distribution category of elderly patients with CRE infection.

Of the 123 elderly patients with CRE infection identified and entered into our study, 79 (64.2%) of these patients were male, and the median age was 82 years. A total of seventy-four patients (60.2%) were designated as the survivor group, while 49 patients (39.8%) were considered as the death group. In the death group, the median (IQR) age was 82 years (78.0–87.5) which was older than that of the survivor group (80.5 years, IQR: 71.0–87.0), and a majority of male patients were observed (31,63.3%), but the differences were not statistically significant (*P* > 0.05).  Table 1 presents detailed demographical and clinical characteristics of all patients. The patients are mainly distributed in ICU (53, 43.1%), followed by the department of emergency medicine (18, 14.6%), department of geriatric (16, 13.0%), and department of cerebral surgical (10, 8.1%) (Figure 1).

### 3.2. Species Distribution of CRE Isolates

A total of one hundred and twenty-three nonrepetitive CRE isolates collected from 123 elderly patients were mainly isolated from sputum (*n* = 83), followed by midstream urine (*n* = 17), pus (*n* = 11), throat swabs (*n* = 8), and blood (*n* = 4). *K. pneumoniae* was the main pathogenic isolates (116, 94.3%), followed by *E. coli* (4, 3.3%), *Serratia marcescens* (*S. marcescens*) (2, 1.6%), and *Klebsiella oxytoca* (*K. oxytoca*) (1, 0.8%).

### 3.3. Antibiotic-Resistant Patterns of Isolates

Table 2 shows the resistant profiles of the 123 CRE isolates against 13 antimicrobial agents, with the prevalence of MDR and XDR rated at 100% and 66.7%, respectively. In general, the 123 CRE isolates exhibited a remarkable resistance to most of the antibiotics tested, with more than 95% of the isolates being resistant to cefepime (100%), piperacillin-tazobactam (99.2%), ceftazidime (98.4%), aztreonam (98.4%), imipenem (98.4%), cefoperazone-sulbactam (98.4%), meropenem (97.6%), and levofloxacin (97.6%). However, tigecycline, polymyxin B, imipenem-avibactam, and ceftazidime-avibactam remained remarkable efficacy against CRE isolates, with minimal resistance rates to those drugs being 0.0%, 0.8%, 7.3%, and 8.9%, respectively. In addition, we also found the isolates from the survivor group demonstrated higher resistance to ceftazidime-avibactam and imipenem-avibactam compared with the death group: 14.9% vs. 0.0% and 12.2% vs. 0.0%.

### 3.4. Carbapenemase and *β*-Lactamase Genotypes in *Enterobacterales*

Five carbapenemase genes, namely, *bla*_KPC_, *bla*_IMP_, *bla*_VIM_, *bla*_NDM_, and *bla*_OXA−48_, and three potential ESBL genotypes, namely, *bla*_CTX−M_, *bla*_TEM_, and *bla*_SHV_, were screened in all 123 isolates. All CRE isolates carried only one type of carbapenemase gene. The *bla*_KPC−2_ was observed in 111 (90.2%) isolates and identified as the most common carbapenemase gene type, while *bla*_NDM−5_ and *bla*_IMP−4_ genes were detected in 5 (4.1%) isolates, respectively, and *bla*_OXA−48_ gene in 2 (1.6%) isolates; however, none of clinical CRE isolates harbored *bla*_VIM_. The harboring of carbapenemase genes in different species of CRE tested in this study exhibited various patterns. In *K. pneumoniae* isolates, *bla*_KPC−2_ was found to be the predominant carbapenemase gene, with 109 (94.0%) of isolates harboring *bla*_KPC−2_, while *bla*_NDM−5_, *bla*_OXA−48_, and *bla*_IMP−4_ were found in only a small proportion (1.7%, 1.7%, and 2.6%, respectively) of isolates. In *E. coli*, all isolates harbored the metallo-carbapenemase gene, with *bla*_NDM−5_, and *bla*_IMP−4_ identified in 75.0% (3/4) and 25.0% (1/4) of isolates. While 2 (100%) *S. marcescens* carried *bla*_KPC−2_, and 1 (100%) *K. oxytoca* was found to have *bla*_IMP−4_.

Furthermore, these CRE isolates showed relatively higher rates for carrying *β*-lactamase encoding genes (98.4%, 121/123), and the prevalence of genes was presented as follows: 106 (86.2%) isolates harbored *bla*_CTX−M_, 121 (98.4%) isolates possessed *bla*_TEM_, and 116 (94.3%) isolates carried *bla*_SHV_. In more detail, various ESBL genetic variants were detected, including *bla*_CTX−M−15_ (*n* = 32), *bla*_CTX−M−14_ (*n* = 74), *bla*_SHV−12_ (*n* = 52), and *bla*_SHV−2a_ (*n* = 5). To be noticed, several non-ESBL gene variants were also identified, with *bla*_SHV−11_ (*n* = 59) and *bla*_TEM−1_ (*n* = 116). The details about *β*-lactamase and carbapenemase genotypes among CRE isolates are shown in Table 3.

### 3.5. Distribution of ST among Clinical *K. pneumoniae* Isolates

Seven STs were identified among 116 *K. pneumoniae* isolates using MLST, of which ST11 (73.3%, 85/116) and ST15 (21.6%, 25/116) were the most prevalent STs, while ST198, ST38, ST147, ST37, and ST5132 were detected in only one isolate each (0.86%, 1/116). In addition, there was one isolate that could not match with any ST type in the database.

### 3.6. Analysis of Risk Factors of Mortality for Patients with CRE Infection

The univariable analysis (Table 1) showed CRE-infected patients with chronic kidney disease were prone to develop adverse outcomes (38.8% vs. 21.6%, *P*=0.039). Higher median APACHE II scores (24 vs. 10, *P* < 0.001) and CCIS score (4 vs. 3, *P*=0.004) were observed in the death group. Meanwhile, patients in the death group were more likely to have intervention therapy during hospitalization including mechanical ventilation (61.2% vs. 23.0%, *P* < 0.001), intravenous catheterization (77.6% vs. 43.2%, *P* < 0.001), presence of indwelling gastric intubation (79.6% vs. 58.1%, *P*=0.013), urinary catheterization (81.6% vs. 56.8%, *P*=0.004), and sputum aspiration (93.9% vs. 75.7%, *P*=0.009) than the ones in the survivor group. In addition, patients with the use of carbapenem antibiotics (67.3% vs. 39.2%, *P*=0.002), tigecycline (75.5% vs. 35.1%, *P* < 0.001), oxazolidinones (36.7% vs. 17.6%, *P*=0.017) during hospitalization, and admission to ICU during hospitalization (63.3% vs. 29.7%, *P* < 0.001) were also to develop adverse outcomes. The multivariable logistic regression analysis (Table 1) implicated mechanical ventilation (AOR = 33.607, *P* < 0.001), use of tigecycline during hospitalization (AOR = 5.868, *P*=0.020), and APACHE II score (AOR = 1.305, *P* < 0.001) as independent risk factors associated with worse clinical outcomes, whereas admission to ICU (AOR = 0.046, *P*=0.011) was a protective factor of mortality.

## 4. Discussion

CRE infection is increasingly identified with high mortality rates. Patients infected with *Enterobacteriale* pathogens resistant to carbapenem display a threefold higher mortality rate compared to those infected with carbapenem-susceptible isolates according to meta-analysis [15, 33]. Moreover, it is noteworthy that the mortality rates for particular subcategories with clinical CRE infections exhibited striking variation, which could reach as high as 78% in liver transplant recipients or as less as 17.5% in patients with a long-term acute care hospital [34, 35]. In the study, we found the all-cause mortality rate among elderly patients of 39.8%, which was consistent with previous studies by Gao et al. [36] and Xu et al. [15], with a mortality rate of 40.15% and pooled overall mortality rate of 42.1%, respectively. However, our finding was higher than the multicenter surveillance study which contained 25 hospitals in 14 provinces in China with an overall hospital mortality rate of 33.5% [37], and the discrepancy could be explained by the fact that our study was focused on the elderly patients aged ≥65 years, different from the abovementioned study which embraced all age groups. Since the majority of the elderly patients in the study had underlying diseases (94.3%, 116/123) and underwent longer lengths of stay (27.0 days, 15.0–49.0) in hospital, which exacerbated patients' poor physical state and caused increased mortality.

All 123 CRE isolates in this study presented MDR, particularly against cephalosporins, *β*-lactamase inhibitors, carbapenems, and fluoroquinolones, with resistance rates being more than 95%, which is a crucial consideration in empirical treatment. Notwithstanding, there are three antibiotic agents in our study that continue to exhibit robust in vitro activities against CRE, namely, tigecycline, polymyxin B, and ceftazidime-avibactam. They were identified as the most efficacious for carbapenem-resistant Gram-negative bacterial infections. Avibactam was an extremely efficient *β*-lactamase inhibitor that has a broader spectrum of activity against *β*-lactamase such as classes A and C and some class D enzymes in Ambler classification. A previous study found that avibactam brought down the MIC_90_ values of ceftazidime and imipenem, which reinstated the efficacy of these by more than sixteen-fold significantly [27], it is in line with the finding of this study. Moreover, avibactam has no activity on class B metalloenzymes lacking serine active site [38]. It was confirmed by the results of our study, 11 (8.9%) isolates displayed resistance against ceftazidime-avibactam, of which 10 isolates produced MBL enzymes, including 5 isolates with NDM-5 and 5 isolates with IMP-4. Interestingly, by investigating the previous medical background of patients, one KPC-2-producing CRKP isolated from a patient who had never been exposed to ceftazidime-avibactam also demonstrated resistance to ceftazidime-avibactam. The mechanism of resistance needs to be proved in further research. Fortunately, all of the 11 ceftazidime-avibactam-resistant isolates remained susceptible to polymyxin B; however, we should pay more attention to renal adverse events of polymyxin B in the clinical treatment of elderly patients with CRE infection, such as nephrotoxicity [37]. Tigecycline was found to be the most effective antibiotic with 100.0% susceptibility rate in CRE isolates, but in our study, the use of tigecycline during hospitalization was also a significant predisposing factor for mortality, which increased the risk about 5.8 times higher odds of death, compared with patients who did not use tigecycline (AOR = 5.868, *P* = 0.020). A meta-analysis revealed that patients treated with tigecycline exhibited a higher incidence of adverse events and mortality rates compared to those receiving other antibiotics [39]. On that account, it is imperative for physicians to be cognizant of the implications for treatment and outcome of this occurrence and the pressing necessity for efficacious antimicrobial agents with a positive safety profile. In a nutshell, a more drastic initial prescription involving three “salvage treatments” agents should be launched early with the guidance of experts in infectious disease in medical care where elderly patients possess a high prevalence of CRE. However, elderly patients may have adverse effects of those drugs, it is imperative to explore additional therapeutic alternatives in order to achieve optimal therapeutic outcomes [33].

Production of carbapenemases is a key mechanism of carbapenem resistance in CRE worldwide and served as the main driver for elderly CRE patients in this work to develop resistance against carbapenems. In the present study, 94.0% CRKP isolates produced KPC-2 carbapenemase, with all belonging to ST11. A multicenter surveillance study in China revealed KPC-2-producing ST11 clone of CRKP was disseminating at an alarming rate and being the common type [40]. ST11 clone present a higher resistance rate than non-ST11, and KPC-2-producing ST11 clone was more easily detected in carbapenem-resistant hypervirulent *Klebsiella pneumoniae*, which facilitated the extensive dissemination of ST11 within China, culminating outbreak in the hospital [40, 41]. Consequently, it is imperative to promptly identify strain characteristics and promptly implement decolonization or treatment measures to prevent the outbreak of ST11. Seventy-five percent carbapenem-resistant *Escherichia coli* (CREC) isolates producing NDM-5, which were in line with the reports of previous research conducted by Yawei Zhang [37], with 74.4% of CREC isolates producing NDM-type enzymes, and by Renru Han [8], with NDM-5, the most common type among CREC (75.0%). Although the fact that ESBLs are incapable of hydrolyzing carbapenem antibiotics, they may still display resistance to these antibiotics when accompanied by a deficiency in membrane pore proteins. In addition, almost all (98.4%) isolates with positive carbapenemase genes in the present study showcased several ESBL genes, also observed by other studies [42, 43]. The carbapenem-resistant mechanism of CRE isolates discovered in this study is of great significance for clinicians to select appropriate antibiotics for treatment in clinical practice and then efficiently restore the health of elderly patients with CRE infection.

After taking into account factors that might influence the outcomes of elderly patients with CRE infection, we found, compared with those who survived during the study period, mechanical ventilation attributed to mortality [44, 45], which was also related to CRKP infection [19, 46, 47]. These patients had an approximately 33.6 times higher possibility of death from CRE infection than those in the survivor group without the experience of mechanical ventilation (AOR = 33.607, *P* < 0.001). Mechanical ventilation is an indispensable intervention for patients in severe conditions, who have a higher risk of developing subsequent infections or recurring infections and dying as a result of infectious complications [46]. The APACHE II score was identified as another predictive indicator of mortality in patients with CRE infection, highlighting its significance as a valuable tool for assessing disease severity and predicting outcomes in individuals with CRKP infection [44, 48]. Our study hinted that APACHE II score had a stronger association with mortality of patients (AOR = 1.305, *P* < 0.001), and a timely scale score at an early stage should be conducted in clinical practice. For these reasons, it is crucial to identify the risk factors associated with mortality and comprehend the existing issues in terms of resistance control and reduction in mortality rates of elderly patients infected with CRE. This will enable health care providers to proactively administer more aggressive treatment to those with potentially worse outcomes [43]. Contrary to other findings [49], the multivariable logistic regression analysis revealed that admission to ICU was a protective factor for death in patients with CRE infection rather than a risk factor (AOR = 0.046, *P*=0.011). Because elderly patients had underlying diseases (94.3%, 116/123) and older age was also a predictor of CRKP infection mortality [44], therefore it is reasonable to assume that patients admitted to ICU will generally receive high-quality medical staff and advanced medical facilities, thus improving their chances of survival.

There are several limitations in our study. Firstly, it is a retrospective design in a single center over the course of one year with an inherent limitation that a relatively small sample size with few patients included. Inadequate information and population may be limited to reliably identifying definitive findings. In addition, it is important to note that these results may not be representative of other patients rather than elderly ones or CRE prevalence in different hospital settings. Secondly, the therapeutic regimens for CRE were not taken into account. Thirdly, we do not further detect the other mechanisms of antibiotic resistance. Despite these limitations, the conclusions in our study can be used as the foundation for further research in this field, and further prospective, multicenter clinical trials are warranted.

## 5. Conclusions

Our data demonstrated a high mortality rate among hospitalized elderly patients with CRE infection. Patients with mechanical ventilation, use of tigecycline, and high APACHE II score are associated with mortality of CRE infection, while admission to ICU was a protective factor. All CRE isolates showed MDR. The presence of carbapenemase gene was an important factor responsible for resistance mechanism to carbapenems, of which *bla*_KPC−2_ was the most dominant type. These findings of the current study would help as a baseline information to design and conduct future detailed investigations on mortality rates among elderly patients with CRE infection, so as to provide crucial guidance for clinicians to select appropriate antibiotic agents for treatment and recognize patients with high risk of death, and then improve the management and clinical outcomes of patients.

## Figures and Tables

**Table 1:** Clinical characteristics and multivariable analysis of risk factors for the death of elderly patients with CRE infection.

Characteristics	Total (*N* = 123)	Survivor group (*N* = 74)	Death group (*N* = 49)	Univariable analysis		Multivariable analysis	
				COR (95% CI)	*P* value	AOR (95% CI)	*P* value
Demographics							
Age (years), median (IQR)	82 (72.0–87.0)	80.5 (71.0–87.0)	82.0 (78.0–87.5)	—	0.105		
Male sex, *n* (%)	79 (64.2)	48 (64.9)	31 (63.3)	0.933 (0.440–1.978)	0.856		
Underlying diseases and complications, *n* (%)	116 (94.3)	69 (93.2)	47 (95.9)	1.703 (0.317–9.148)	0.702		
Cardiovascular and cerebrovascular diseases, *n* (%)	90 (73.2)	53 (71.6)	37 (75.5)	1.222 (0.536–2.786)	0.634		
Chronic lung diseases, *n* (%)	16 (13.0)	10 (13.5)	6 (12.2)	0.893 (0.302–2.639)	0.838		
Chronic kidney diseases, *n* (%)	35 (28.5)	16 (21.6)	19 (38.8)	2.296 (1.034–5.098)	0.039^∗^		
Biliary tract diseases, *n* (%)	22 (17.9)	11 (14.9)	11 (22.4)	1.658 (0.656–4.192)	0.283		
Solid tumors, *n* (%)	21 (17.1)	12 (16.2)	9 (18.4)	1.163 (0.449–3.010)	0.756		
Diabetes mellitus, *n* (%)	58 (47.2)	36 (48.6)	22 (44.9)	0.860 (0.417–1.775)	0.683		
Hypertensions, *n* (%)	68 (55.3)	36 (48.6)	32 (65.3)	1.987 (0.944–4.182)	0.069		
APACHE II score, median (IQR)	14.0 (10.0–23.0)	10 (8.0–14.3)	24 (20.0–31.5)	—	<0.001⁣^∗^	1.305 (1.161–1.468)	<0.001⁣^∗^
CCIS, median (IQR)	3 (2–5)	3 (1–4)	4 (2–6)	—	0.004⁣^∗^		
LOS (days), median (IQR)	27 (15–49)	34 (16–50)	29 (14–43)	—	0.282		
≤14, *n* (%)	28 (22.8)	14 (18.9)	14 (28.6)	1.714 (0.733–4.011)	0.211		
15–28, *n* (%)	34 (27.6)	22 (29.7)	12 (24.5)	0.767 (0.338–1.741)	0.525		
≥29, *n* (%)	61 (49.6)	38 (51.4)	23 (46.9)	0.838 (0.407–1.727)	0.632		
Admission to intensive care unit during hospitalization, *n* (%)	53 (43.1)	22 (29.7)	31 (63.3)	4.071 (1.894–8.751)	<0.001⁣^∗^	0.046 (0.004–0.496)	0.011⁣^∗^
Nosocomial infection, *n* (%)	90 (73.2)	56 (75.7)	34 (69.4)	0.729 (0.325–1.633)	0.441		
Health care exposures within 30 days, *n* (%)	45 (36.6)	27 (36.5)	18 (36.7)	1.011 (0.478–2.138)	0.978		
Intervention therapy during hospitalization, *n* (%)	115 (93.5)	66 (89.2)	49 (100)	—	0.021⁣^∗^		
Surgery, *n* (%)	17 (13.8)	11 (14.9)	6 (12.2)	0.799 (0.275–2.324)	0.680		
Mechanical ventilation, *n* (%)	47 (38.2)	17 (23.0)	30 (61.2)	5.294 (2.403–11.661)	<0.001⁣^∗^	33.607 (4.176–270.463)	<0.001⁣^∗^
Central intravenous catheter, *n* (%)	70 (56.9)	32 (43.2)	38 (77.6)	4.534 (2.011–10.228)	<0.001⁣^∗^		
Gastric intubation, *n* (%)	82 (66.7)	43 (58.1)	39 (79.6)	2.812 (1.221–6.475)	0.013⁣^∗^		
Urinary catheterization, *n* (%)	82 (66.7)	42 (56.8)	40 (81.6)	3.386 (1.437–7.978)	0.004⁣^∗^		
Sputum aspiration, *n* (%)	102 (82.9)	56 (75.7)	46 (93.9)	4.929 (1.366–17.779)	0.009⁣^∗^		
Antibiotic exposure before hospitalization (within 30 days), *n* (%)	64 (52.0)	39 (52.7)	25 (51.0)	0.935 (0.454–1.925)	0.855		
Medication therapy during hospitalization, *n* (%)	117 (95.1)	68 (91.9)	49 (100)	—	0.080		
Glycopeptides, *n* (%)	25 (20.3)	14 (18.9)	11 (22.4)	1.241 (0.510–3.015)	0.634		
Oxazolidinones, *n* (%)	31 (25.2)	13 (17.6)	18 (36.7)	2.725 (1.183–6.274)	0.017⁣^∗^		
Chloramphenicols, *n* (%)	20 (16.3)	10 (13.5)	10 (20.4)	1.641 (0.627–4.297)	0.310		
*β*-lactams/*β*-lactamase inhibitors, *n* (%)	65 (52.8)	36 (48.6)	29 (59.2)	1.531 (0.738–3.175)	0.252		
Tigecyclines, *n* (%)	63 (51.2)	26 (35.1)	37 (75.5)	5.692 (2.539–12.761)	<0.001⁣^∗^	5.868 (1.318–26.130)	0.020⁣^∗^
3rd or 4th generation cephalosporins, *n* (%)	45 (36.6)	24 (32.4)	21 (42.9)	1.563 (0.741–3.296)	0.240		
1st or 2nd generation cephalosporins, *n* (%)	10 (8.1)	5 (6.8)	5 (10.2)	1.568 (0.429–5.731)	0.517		
Carbapenems, *n* (%)	62 (50.4)	29 (39.2)	33 (67.3)	3.200 (1.500–6.829)	0.002⁣^∗^		
Fluoroquinolones, *n* (%)	80 (65.0)	42 (56.8)	38 (77.6)	2.632 (1.167–5.937)	0.018⁣^∗^		
Aminoglycosides, *n* (%)	35 (28.5)	25 (33.8)	10 (20.4)	0.503 (0.216–1.170)	0.107		

⁣^∗^A significant value, *P* < 0.05. CRE: carbapenem-resistant *Enterobacterales*; COR: crude odds ratio; AOR: adjusted odds ratio; CI: confidence interval; IQR: interquartile range; APACHE: acute physiology and chronic health evaluation. CCIS: Charlson comorbidity index score. LOS: Length of hospital stay.

**Table 2:** General AST patterns of *Enterobacterales* isolates from elderly patients.

Drug	Survivor group (*n* = 74)				Death group (*n* = 49)				Total (*n* = 123)		
	MIC range (*μ*g/mL)	MIC_50_ (*μ*g/mL)	MIC_90_ (*μ*g/mL)	Resistant *n* (%)	MIC range (*μ*g/mL)	MIC_50_ (*μ*g/mL)	MIC_90_ (*μ*g/mL)	Resistant *n* (%)	MIC_50_ (*μ*g/mL)	MIC_90_ (*μ*g/mL)	Resistant *n* (%)
CAZ	8–>128	>128	>128	72 (97.3)	16–>128	>128	>128	49 (100)	>128	>128	121 (98.4)
CZA	0.5/4–>128/4	2/4	>128/4	11 (14.9)	0.5/4–8/4	4/4	8/4	0 (0.0)	4/4	8/4	11 (8.9)
FEP	32–>128	>128	>128	74 (100)	16–>128	>128	>128	49 (100)	>128	>128	123 (100)
AZT	1–>128	>128	>128	72 (97.3)	128–>128	>128	>128	49 (100)	>128	>128	121 (98.4)
TZP	32–>512/4	>512/4	>512/4	73 (98.6)	128/4–>512/4	512/4	512/4	49 (100)	>512/4	>512/4	122 (99.2)
IPM	1–>32	32	>32	72 (97.3)	8–>32	>32	>32	49 (100)	32	>32	121 (98.4)
IPM/AV	0.25/4–>32/4	0.5/4	4/4	9 (12.2)	0.25/4−2/4	0.25/4	1/4	0 (0.0)	0.25/4	2/4	9 (7.3)
MEM	0.5–>32	>32	>32	71 (95.9)	16–>32	>32	>32	49 (100)	>32	>32	120 (97.6)
AMK	0.5–>512	512	>512	49 (66.2)	4–>512	>512	>512	36 (73.5)	>512	>512	85 (69.1)
PB	0.25–>32	0.5	1	1 (1.4)	0.25–2	0.5	1	0 (0.0)	0.5	1	1 (0.8)
TGC	0.125–2	1	2	0 (0.0)	0.25–2	1	2	0 (0.0)	1	2	0 (0.0)
LVX	1–>64	32	64	71 (95.9)	8–>64	64	>64	49 (100)	32	64	120 (97.6)
SCF	32/16–>512/128	>256/128	>256/128	73 (98.6)	32/16–>256/128	>256/128	>256/128	48 (98.0)	>256/128	>256/128	121 (98.4)

CAZ: ceftazidime; CZA: ceftazidime-avibactam; FEP: cefepime; AZT: aztreonam; TZP: piperacillin-tazobactam; IPM: imipenem; IPM/AV: imipenem-avibactam; MEM: meropenem; AMK: amikacin; PB: polymyxin B; TGC: tigecycline; LVX: levofloxacin; SCF: cefoperazone-sulbactam; AST: antimicrobial susceptibility testing; MIC: minimum inhibitory concentration.

**Table 3:** Carbapenemase and *β*-lactamase genotypes among carbapenem-resistant *Enterobacterales*.

Genes	Total (*n* = 123), *n* (%)	*K. pneumoniae* (*n* = 116), *n* (%)	*E. coli* (*n* = 4), *n* (%)	*S. marcescens* (*n* = 2), *n* (%)	*K. oxytoca* (*n* = 1), *n* (%)
Carbapenemase genes					
*bla*_KPC−2_	111 (90.2)	109 (94.0)	0 (0.0)	2 (100)	0 (0.0)
*bla*_NDM−5_	5 (4.1)	2 (1.7)	3 (75.0)	0 (0.0)	0 (0.0)
*bla*_OXA−48_	2 (1.6)	2 (1.7)	0 (0.0)	0 (0.0)	0 (0.0)
*bla*_IMP−4_	5 (4.1)	3 (2.6)	1 (25.0)	0 (0.0)	1 (100)
*bla*_VIM_	0 (0.0)	0 (0.0)	0 (0.0)	0 (0.0)	0 (0.0)
*β*-lactamase genes					
*bla*_CTX−M_					
*bla*_CTX−M−14_	74 (60.2)	74 (63.8)	0 (0.0)	0 (0.0)	0 (0.0)
*bla*_CTX−M−15_	32 (26.0)	28 (24.1)	4 (100)	0 (0.0)	0 (0.0)
*bla*_TEM_					
*bla*_TEM − 1^∗^_	121 (98.4)	116 (100)	4 (100)	0 (0.0)	1 (100)
*bla*_SHV_					
*bla*_SHV − 11^∗^_	59 (48.0)	59 (50.9)	0 (0.0)	0 (0.0)	0 (0.0)
*bla*_SHV−12_	52 (42.3)	52 (44.8)	0 (0.0)	0 (0.0)	0 (0.0)
*bla*_SHV−2a_	5 (4.1)	5 (4.3)	0 (0.0)	0 (0.0)	0 (0.0)

⁣^∗^Non-ESBL encoding gene.

## Data Availability

The data used to support the findings of this study are included within the article and supplementary materials. Further inquiries are available from the corresponding authors upon reasonable request.

## Supplementary Materials

Supplementary Materials Details of PCR to detect carbapenemase and ESBL genes in *Enterobacterales*. Table S1: primers for detection of antibiotic genes.
