# Supplementary material for: Predictors of Mortality, Drug Resistance, and Determinants among Carbapenem-Resistant Enterobacterales Infections in Chinese Elderly Patients
Source: Can J Infect Dis Med Microbiol. 2024 Aug 28;2024:5459549. doi: 10.1155/2024/5459549 (PMC11374419; doi:10.1155/2024/5459549)
Supplement: Supplementary Materials — Details of PCR to detect carbapenemase and ESBL genes in Enterobacterales. Table S1: primers for detection of antibiotic genes. [file 5459549.f1.doc]

# Predictors of mortality, drug resistance and determinants among carbapenem-resistant *Enterobacteriales* infections in Chinese elderly patients

Yufei Zhang^1^, Chengyun Zou^2^, Jie Qin^1^, Muyi Li^1^, Xing Wang^3^, Tian Wei^1^ and Haiying Wang^1^

Correspondence should be addressed to Tian Wei; [1347781823@qq.com](mailto:1347781823@qq.com) and Haiying Wang; [liang19961111@shutcm.edu.cn](mailto:liang19961111@shutcm.edu.cn)

### Details of PCR to detect carbapenemase and ESBLs genes in *Enterobacteriales*

The PCR was performed in a final volume of 10ul containing 5ul 2 x Hot StarTaq multiplex PCR Master Mix (TIANGEN, Beijing, China), 0.5ul of each primer (0.2uM), 1.0ul of template DNA (300ng), and 3ul of nuclease-free water. The oligonucleotide sequence of primers of carbapenemase and ESBLs genes were shown in supplementary Table 1. The PCR cycling parameters for both reactions were: initial denaturation at 95°C for 15 minutes followed by 35 cycles each of denaturation at 94°C for 30s, annealing at 60°C for 30s, extension at 72°C for 1 minute, and final extension at 72°C for 10 minutes. The PCR products were visualized by performing gel-electrophoresis in 1% agarose gel after staining in GeneGreen (TIANGEN, Beijing, China) with the aid of a gel imaging system (Clinx Science Instruments, China). A 2000bp ladder molecular weight marker (TaKaRa, Japan) was used to measure the molecular weight of amplified products.

Supplementary Table 1: Primers for detection of antibiotic genes.

| Gene | Primer | Oligonucleotide sequence (5′-3′) | Size (bp) | Reference |
| --- | --- | --- | --- | --- |
| *KPC* | KPC-F | AGGACTTTGGCGGCTCCAT | 720 | [1] |
|  | KPC-R | TCCCTCGAGCGCGAGTCTA |  |  |
| *NDM* | NDM-F | GGTTTGGCGATCTGGTTTTC | 621 | [2] |
|  | NDM-R | CGGAATGGCTCATCACGATC |  |  |
| *OXA-48* | OXA-48F | GCTTGATCGCCCTCGATT | 281 | [3] |
|  | OXA-48R | GATTTGCTCCGTGGCCGAAA |  |  |
| *IMP* | IMP-F | CTACCGCAGCAGAGTCTTTG | 587 | [4] |
|  | IMP-R | AACCAGTTTTGCCTTACCAT |  |  |
| *VIM* | VIM-F | GATGGTGTTTGGTCGCATA | 390 | [2] |
|  | VIM-R | CGAATGCGCAGCACCAG |  |  |
| *CTX-M* | CTX-M-F | ATGTGCAGYACCAGTAARGTKATGGC | 593 | [5] |
|  | CTX-M-R | TGGGTRAARTARGTSACCAGAAYCAGCGG |  |  |

Supplementary Table 1: Continued.

| Gene | Primer | Oligonucleotide sequence (5′-3′) | Size (bp) | Reference |
| --- | --- | --- | --- | --- |
| *TEM* | TEM-F | TCGCCGCATACACTATTCTCAGAATGA | 445 | [5] |
|  | TEM-R | ACGCTCACCGGCTCCAGATTTAT |  |  |
| *SHV* | SHV-F | ATGCGTTATATTCGCCTGTG | 747 |  |
|  | SHV-R | TGCTTTGTTATTCGGGCCAA |  |  |

## Reference

1. Qin, X.; Yang, Y.; Hu, F.; Zhu, D. Hospital clonal dissemination of *Enterobacter aerogenes* producing carbapenemase KPC-2 in a Chinese teaching hospital. *J Med Microbiol* **2014**, *63*, 222-228, doi:10.1099/jmm.0.064865-0.

2. Poirel, L.; Walsh, T.R.; Cuvillier, V.; Nordmann, P. Multiplex PCR for detection of acquired carbapenemase genes. *Diagn Microbiol Infect Dis* **2011**, *70*, 119-123, doi:10.1016/j.diagmicrobio.2010.12.002.

3. Dallenne, C.; Da Costa, A.; Decre, D.; Favier, C.; Arlet, G. Development of a set of multiplex PCR assays for the detection of genes encoding important beta-lactamases in *Enterobacteriaceae*. *J Antimicrob Chemother* **2010**, *65*, 490-495, doi:10.1093/jac/dkp498.

4. Senda, K.; Arakawa, Y.; Ichiyama, S.; Nakashima, K.; Ito, H.; Ohsuka, S.; Shimokata, K.; Kato, N.; Ohta, M. PCR detection of metallo-beta-lactamase gene (*bla*_IMP_) in gram-negative rods resistant to broad-spectrum beta-lactams. *J Clin Microbiol* **1996**, *34*, 2909-2913, doi:10.1128/jcm.34.12.2909-2913.1996.

5. Monstein, H.J.; Ostholm-Balkhed, A.; Nilsson, M.V.; Nilsson, M.; Dornbusch, K.; Nilsson, L.E. Multiplex PCR amplification assay for the detection of *bla*_SHV_, *bla*_TEM_ and *bla*_CTX-M_ genes in *Enterobacteriaceae*. *APMIS* **2007**, *115*, 1400-1408, doi:10.1111/j.1600-0463.2007.00722.x.
